# The impact of implementation of a national smoke-free prisons policy on indoor air quality: results from the Tobacco in Prisons study

**DOI:** 10.1136/tobaccocontrol-2018-054895

**Published:** 2019-05-07

**Authors:** Sean Semple, Ruaraidh Dobson, Helen Sweeting, Ashley Brown, Kate Hunt, on behalf of the Tobacco in Prisons (TIPs) research team

**Affiliations:** 1 Institute of Social Marketing, University of Stirling, Stirling, UK; 2 MRC/CSO Social and Public Health Sciences Unit, University of Glasgow, Glasgow, UK

**Keywords:** correctional facilities, SHS, ETS, work, PM_2.5_

## Abstract

**Objective:**

To determine secondhand smoke (SHS) concentrations in prisons during the week of implementation of a new, national prisons smoke-free policy.

**Design:**

Repeated measurement of SHS concentrations immediately before and after implementation of smoke-free policies across all 15 prisons in Scotland, and comparison with previously gathered baseline data from 2016.

**Methods:**

Fine particulate matter (PM_2.5_) measurements at a fixed location over a continuous 6-day period were undertaken at the same site in each prison as previously carried out in 2016. Outdoor air quality data from the nearest local authority measurement station were acquired to determine the contribution of outdoor air pollution to indoor prison measurement of PM_2.5_.

**Results:**

Air quality improved in all prisons comparing 2016 data with the first full working day postimplementation (overall median reduction −81%, IQR −76% to −91%). Postimplementation indoor PM_2.5_ concentrations were broadly comparable with outdoor concentrations suggesting minimal smoking activity during the period of measurement.

**Conclusions:**

This is the first evaluation of changes in SHS concentrations across all prisons within a country that has introduced nationwide prohibition of smoking in prisons. All prisons demonstrated immediate substantial reductions in PM_2.5_ following policy implementation. A smoke-free prisons policy reduces the exposure of prison staff and prisoners to SHS.

## Introduction

Secondhand smoke (SHS) is a serious indoor air pollutant linked to many illnesses, including cardiovascular disease, cancer and chronic obstructive pulmonary disease.^1^ Smoking bans in indoor environments reduce exposure to SHS^2^ and improve health.^3 4^ SHS exposure has been a concern for workers who are or were occupationally exposed, for example, restaurant staff^5^ and airline cabin crew.^6^

Until recently, prisons had partial exemption from the Smoking, Health and Social Care (Scotland)Act 2005,^7^ which banned smoking in most enclosed public spaces. Partly in response to the perceived social importance of smoking in prison culture,^8^ prisoners were permitted to smoke in their cells with the doors closed. Prisons were, thus, one of the few UK workplaces in which staff were exposed to SHS. Research by the Tobacco in Prisons (TIPs) study team in 2016 on indoor air quality demonstrated high concentrations of SHS in prison hallways and other areas where staff could be exposed during their work.^9^ These results informed policy development with the Scottish Prison Service’s Chief Executive calling the data a ‘wake-up call’ to action in 2017^10^ when he announced that a new policy would be implemented on Friday 30 November 2018 to prohibit smoking throughout all prisons in Scotland, both indoors and outdoors. This rule change follows the implementation of smoking restrictions in prison systems elsewhere in the UK and internationally (eg, New Zealand, parts of Australia, Canada and parts of the USA).

Although the policy was set to change on this date, this did not necessarily mean smoking would immediately stop. Results from a previous phase of TIPs indicated that a majority of prisoners viewed the planned ban unfavourably, with less than a quarter of those surveyed agreeing that ‘prison smoking bans are a good idea’.^8^ Tobacco was on sale in prisons until 2 weeks before the implementation date, and it was considered plausible that prisoners might stockpile tobacco to smoke after the ban was implemented. It was, therefore, of interest to measure the impact of the new policy immediately after its introduction.

This study evaluates and quantifies the impact of this policy change on measurable SHS within prisons immediately before and after the ban, in a manner directly comparable to our previous research on SHS in Scotland’s prisons.^9^

## Methods

### Quantification of SHS in prisons

Fine particulate matter (PM_2.5_) is widely used as a proxy measurement for SHS in indoor air^11^ as it is simple to measure and, where smoking occurs, is closely correlated with SHS concentrations.

Dylos DC1700 air quality monitors (Dylos Corp., Riverside, CA, USA) were used to measure PM_2.5_ in each prison. These have been validated for this purpose.^12^ Dylos-reported particle number concentrations were converted to mass concentrations of PM_2.5_ using an equation described previously.^13^ Each Dylos was individually calibrated against a TSI SidePak AM510 (TSI, Shoreview, MN, USA) using a calibration factor of 0.295 in a chamber experiment where fresh SHS was generated from a smouldering cigarette. The calibration factor derived for each individual Dylos device was then applied to the calculated mass concentration to produce the final value.

As previously,^9^ staff in each prison were trained to operate and monitor the Dylos devices and tasked with installing the instrument, switching it on and off at the start and end of the measurement period. Devices were placed in the same fixed location within a residential hall in each prison as used during the measurements in 2016,^9^ to ensure comparability.

Monitoring was scheduled for 6 days between 09:00 Wednesday 28 November and 09:00 Tuesday 4 December 2018. This timing was chosen to allow observation of the period immediately before and after the ban was introduced (00:01 Friday 30 November), utilising the full extent of the Dylos’ memory capacity.

Dylos data were downloaded using the Dylos Logger software. Hourly outdoor PM_2.5_ data for the whole measurement period were also downloaded from the nearest environmental monitoring station to each prison, which provided gravimetric PM_2.5_ concentration data (via www.scottishairquality.co.uk).

### Statistical analysis

Arithmetic mean calculated PM_2.5_ mass concentrations from the first, preban day of measurement (09:00 28 November to 08:59 29 November) were compared with the last, postban day of measurement (09:00 3 December to 08:59 4 December) to determine the effect of policy implementation. Overall 6-day arithmetic mean concentrations measured in each prison in 2016 were compared with the overall 6-day mean concentrations in 2018, and to the individual 24 hours preban and postban period mean concentrations from this phase of measurement. Arithmetic means were preferred for comparability with previous studies on PM_2.5_ concentrations in prisons^9 14^; though to take account of the likely skewed distribution of exposure data of this nature, we also present data as medians in online supplementary table 1. To test the significance of any change, paired Wilcoxon signed-rank tests were conducted between the preban and postban results, and between 2016 and 2018 6-day mean concentrations.

10.1136/tobaccocontrol-2018-054895.supp1 Supplementary data

Statistical analysis, including conversion to mass concentration, was conducted using Microsoft Excel (Office 2016) and IBM SPSS statistics software (Version 23.0).

## Results

### Data integrity

Measurements gathered over 114 000 min of viable data; mean duration of measurement was 7620 min (range 2606–8648). Three prisons (#1, #8 and #11) had interrupted or shortened measurement periods (details provided in the footnote to online supplementary table 2).

10.1136/tobaccocontrol-2018-054895.supp2 Supplementary data

### Comparison of SHS-related PM_2.5_ in prisons immediately preban and postban

PM_2.5_ levels declined substantially in every prison between (2018) preban and postban periods from a median of 12.7 to 4.7 µg/m^3^ (median decline of −46%; IQR −31% to −65%) (p<0.001). Full results from each prison are presented in online supplementary table 2.

The median distance from an outdoor monitor to the prison to which it was compared was 14.5 km (range 1.4–77 km; 11/15<25 km). PM_2.5_ concentrations in ambient outdoor air were generally low over the period of measurement (median 5.0 µg/m^3^, IQR 4.7–6.4). To determine the impact of outdoor pollution on indoor monitoring results, the mean outdoor PM_2.5_ over the period was subtracted from mean indoor PM_2.5_ at each prison. The median of these corrected mean concentrations was 0.03 µg/m^3^ (range −3 to 8 µg/m^3^), suggesting minimal PM_2.5_ emission associated with smoking within the prisons during the period of measurement.

### Comparison of SHS-related PM_2.5_ between 2016 and 2018

**Figure 1:**
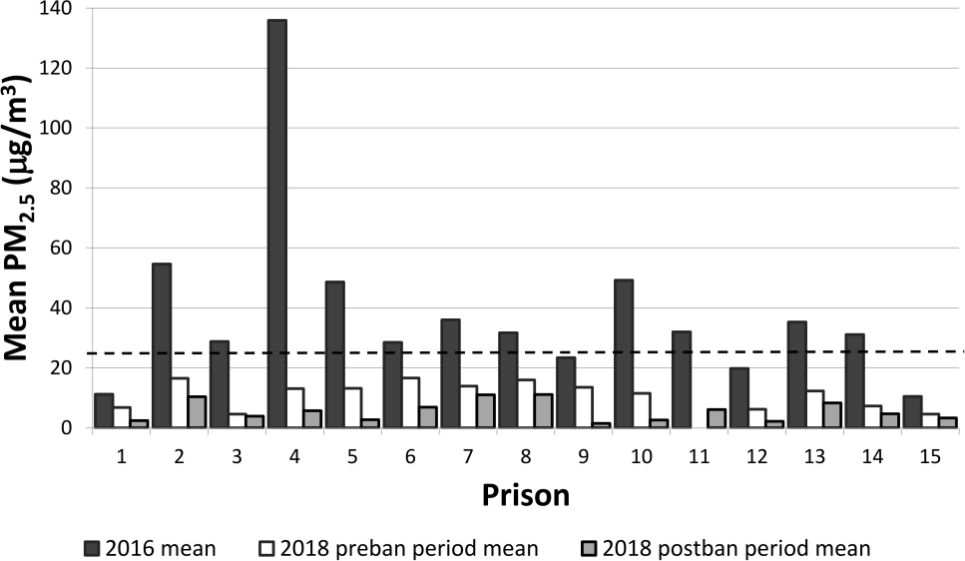
Mean PM_2.5_ from 6 days of measurement in 2016, 1-day preban (November 2018) and 1-day postban (December 2018). Every prison with usable data saw declines across each period. The dashed line represents the WHO guideline limit for 24 hours exposure to PM_2.5_ (25 µg/m^3^). No 2018 preban data are available for prison 11. PM_2.5_, fine particulate matter.

Mean concentrations from the 2016 measurements^9^ and the 2018 preban and postban periods are shown in figure 1 for each prison. Mean concentration declined in every prison between 2016 and 2018, from a median of 31.7 µg/m^3^ (IQR 23.4–48.6) to 5.8 µg/m^3^ (IQR 4.0–10.7) (p=0.001). Comparing the 2016 values with the 24 hour measurement made on Monday 3rd December 2018 across the 15 prisons shows an overall median reduction in PM_2.5_ concentrations of 81% (IQR 76%–91%).

## Discussion

To the best of our knowledge, this is the first study to report objectively measured effects on indoor air quality of a smoking ban in all prisons within a country immediately before and after policy implementation, with levels preceding the announcement of a ban. The results show that the anticipation and introduction of the smoking ban in Scotland’s prisons had a significant and substantial effect on indoor PM_2.5_ concentrations, suggesting reduced prison staff and prisoner SHS exposure.

There was a substantial decline between the 2016 measurements and the 2018 measurements immediately preban. This may reflect increased enforcement of the previous smoking policy indoors (only in prison cells with doors closed), as the previous work^9^ had increased awareness of the risk to staff and prisoners from SHS exposure. Additionally, it is likely that removal of tobacco from prisons (no longer available for purchase in prisons’ canteens; from w/c 19 November) will have reduced prisoner smoking levels, with some having run out of tobacco before the implementation date. Provision of smoking cessation assistance together with the availability of rechargeable vaping devices to eligible prisoners in the period leading up to the 30 November may also have contributed to the measured improvements in air quality.

The improvement in indoor air quality reported in this study was comparable to that seen in previous research. In a study of North Carolina’s prison system,^14^ researchers measured PM_2.5_ concentrations before and after a ban in six prisons, observing a decline of 77% following the introduction of the policy, while another study in one maximum security prison in New Zealand^15^ suggested that PM_2.5_ concentrations declined by an average of 57% following a nationwide ban. Our results are in contrast to a study of a single Australian prison that implemented a smoking ban.^16^ That work reported increased PM_2.5_ concentrations postban and suggested this was due to clandestine smoking taking place.

### Strengths and weaknesses

In addition to capturing data throughout Scotland’s prison estate, a particular strength of this study was our measurement for a full 6-day period, providing directly comparable data for 24 hours periods rather than snapshots from shorter periods when instruments were installed.^17^ Using the nearest government air quality measurement sites enabled us to compare ambient PM_2.5_ concentrations with those in each prison.

The Dylos instruments used in this study were calibrated against another optical monitor, a TSI SidePak, using a previously determined correction factor for SHS (0.295), in the same manner as in a previous paper.^9^ The authors did not directly calibrate the SidePak using a gravimetric method of measuring PM_2.5_ before conducting these calibrations but the SidePak is factory calibrated by the manufacturer against known PM_2.5_ concentrations.

As this study took place during the implementation week, to assess immediate impacts, a later phase of the TIPs research project will measure PM_2.5_ concentrations in prisons 6 months postban, to determine whether the low levels of SHS, observed immediately post-implementation, continue.

## Conclusions

The study demonstrates widespread improvements in prison air quality as a result of the smoke-free policy. The exposure of prison staff and prisoners to SHS is likely to be considerably reduced as a result of the implementation of this policy.

**What this paper adds**

- This is the first evaluation of changes in secondhand smoke concentrations across all prisons within a country that has introduced nationwide prohibition of smoking in prisons.
- The study demonstrates widespread improvements in prison air quality following the implementation of a total smoking ban.
- All 15 prisons demonstrated substantial and statistically significant reductions in fine particulate matter concentrations in the week when the smoke-free policy was implemented compared with previous directly comparable measurements made in 2016.
