# Supplementary material for: The impact of implementation of a national smoke-free prisons policy on indoor air quality: results from the Tobacco in Prisons study
Source: Tob Control. 2019 May 7;29(2):234–6. doi: 10.1136/tobaccocontrol-2018-054895 (PMC7036294; doi:10.1136/tobaccocontrol-2018-054895)
Supplement: Supplementary data [file tobaccocontrol-2018-054895supp001.pdf]

Supplementary table 1: Results of $PM_{2.5}$ monitoring by prison (median concentrations)

Prison	Duration (minutes)	Median 6-day (2018) $PM_{2.5}$ (IQR) (μg/m³)	Outdoor mean $PM_{2.5}$ (μg/m³)	Median pre-ban (Nov18) period $PM_{2.5}$ (μg/m³)	Median post-ban (Dec18) period $PM_{2.5}$ (μg/m³)	Change after ban (%)	Change between 2016 and post-ban (Dec18) (%)
**1**	3469	2.3 (1.5-4.3)	4.3	5.4*	2.3*	-58	-70
**2**	8632	15.8 (10.9-18.7)	6.9	16.3	9.2	-44	-61
**3**	7661	3.5 (2.4-5)	5.0	4.1	3.2	-21	-86
**4**	8648	6.6 (2.5-13.2)	4.9	12.2	2.2	-82	-80
**5**	8641	3.3 (2.2-4.4)	6.7	11.3	2.6	-77	-90
**6**	8635	13.1 (6.8-16.3)	6.4	16.1	5.8	-64	-50
**7**	8640	11.4 (7.4-15.7)	5.8	14.0	10.9	-23	-71
**8**	7244	9.4 (4.9-13.3)	3.9	16.4†	9.5†	-42	-72
**9**	7322	2.9 (1.7-5.8)	4.9	14.8	1.5	-90	-88
**10**	8637	4.6 (2.7-10)	4.7	12.6	2.3	-82	-84
**11**	2606	4 (2.2-7.5)	5.3	‡	5.2	‡	-89
**12**	8640	2.9 (1.9-4.9)	6.9	5.1	2.0	-62	-85
**13**	8636	11.5 (6.2-14.9)	5.0	13.1	7.4	-43	-65
**14**	8267	4.6 (3-8.1)	5.8	6.6	4.2	-36	-87
**15**	8625	3 (2.1-4.8)	4.3	3.9	2.0	-49	-64
**Median**		4.6 (3-11.4)	5.0	12.4	3.2	-53.5	-80

* *Comparison of pre- and post-ban data at prison 1 used the periods from 09:03 to 22:06 on 28 November and on 3 November.*

† *Comparison of pre- and post-ban data at prison 8 used the periods from 09:00 to 23:59 on 29 November and on 3 November.*

‡ *Prison 11's monitor started recording after 14:00 on 2 December (post-ban) so no pre-ban data is available for this site.*
