# Supplementary material for: The impact of implementation of a national smoke-free prisons policy on indoor air quality: results from the Tobacco in Prisons study
Source: Tob Control. 2019 May 7;29(2):234–6. doi: 10.1136/tobaccocontrol-2018-054895 (PMC7036294; doi:10.1136/tobaccocontrol-2018-054895)
Supplement: Supplementary data [file tobaccocontrol-2018-054895supp002.pdf]

Supplementary table 2: Overall results of $PM_{2.5}$ monitoring by prison (arithmetic mean concentrations)

Prison	Duration (minutes)	Mean 6-day (2018) $PM_{2.5}$ (SD) (μg/m^3)	Outdoor mean $PM_{2.5}$ (μg/m^3)	Pre-ban (Nov18) period $PM_{2.5}$ (μg/m^3)	Post-ban (Dec18) period $PM_{2.5}$ (μg/m^3)	Change after ban (%)	Change between 2016 and post-ban (Dec18) (%)
1	3469	3.5 (3.5)	4.3	6.8*	2.4*	-65	-79
2	8632	14.6 (5.2)	6.9	16.5	10.4	-37	-81
3	7661	3.9 (2.0)	5.0	4.6	3.9	-14	-86
4	8648	10.5 (21.4)	4.9	13.1	5.7	-56	-96
5	8641	4.9 (5.2)	6.7	13.2	2.7	-80	-94
6	8635	12.1 (6.7)	6.4	16.6	6.9	-59	-76
7	8640	11.3 (4.8)	5.8	13.9	11.0	-21	-69
8	7244	9.4 (6.2)	3.9	16.0†	11.1†	-31	-65
9	7322	4.8 (4.8)	4.9	13.5	1.5	-89	-94
10	8637	6.4 (4.6)	4.7	11.5	2.6	-77	-95
11	2606	5.1 (3.6)	5.3	‡	6.1	‡	-81
12	8640	3.8 (3.8)	6.9	6.2	2.2	-65	-89
13	8636	10.7 (4.8)	5.0	12.3	8.3	-32	-76
14	8267	5.8 (3.9)	5.8	7.3	4.7	-35	-85
15	8625	4 (4.8)	4.3	4.6	3.3	-28	-69
Median		5.8	5.0	12.7	4.7	-46.5	-81

* *Comparison of pre- and post-ban data at prison 1 used the periods from 09:03 to 22:06 on 28 November and on 3 November.*

† *Comparison of pre- and post-ban data at prison 8 used the periods from 09:00 to 23:59 on 29 November and on 3 November.*

‡ *Prison 11's monitor started recording after 14:00 on 2 December (post-ban) so no pre-ban data is available for this site.*
